# Connecting Diffraction Experiments and Network Analysis Tools for the Study of Hydrogen-Bonded Networks

**DOI:** 10.1021/acs.jpcb.2c07740

**Published:** 2023-03-30

**Authors:** Imre Bakó, Szilvia Pothoczki, László Pusztai

**Affiliations:** † Research Centre for Natural Sciences, Budapest H-1121, Hungary; ‡ Wigner Research Centre for Physics, Konkoly Thege út 29-33, Budapest H-1121, Hungary

## Abstract

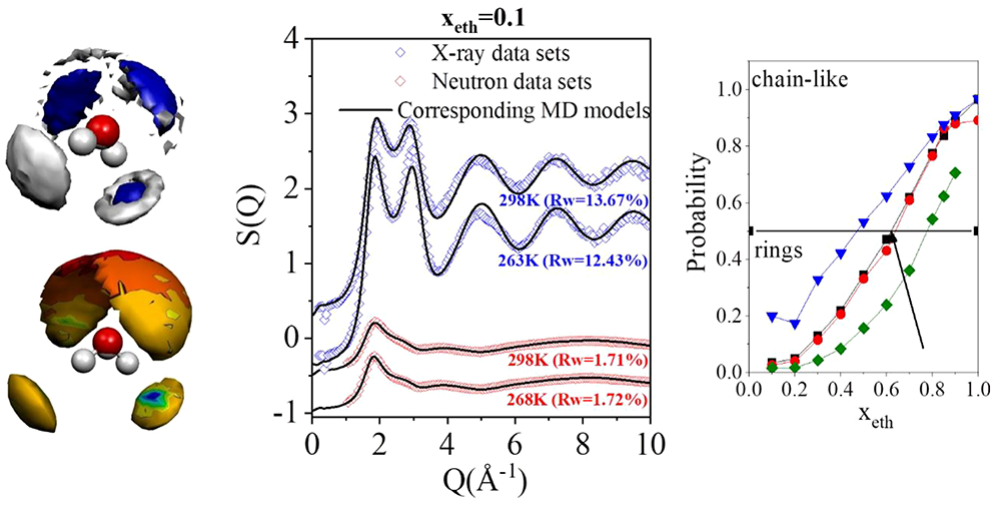

A self-consistent scheme is presented that is applicable for revealing details of the microscopic structure of hydrogen-bonded liquids, including the description of the hydrogen-bonded network. The scheme starts with diffraction measurements, followed by molecular dynamics simulations. Computational results are compared with the experimentally accessible information on the structure, which is most frequently the total scattering structure factor. In the case of an at least semiquantitative agreement between experiment and simulation, sets of particle coordinates from the latter may be exploited for revealing nonmeasurable structural details. Calculations of some properties concerning the hydrogen-bonded network are also described, in the order of increasing complexity: starting with the definition of a hydrogen bond, first and second neighborhoods are described via spatial correlations functions. Attention is then turned to cyclic and noncyclic hydrogen-bonded clusters, before cluster size distributions and percolation are discussed. We would like to point out that, as a result of applying the novel protocol, these latter, rather abstract, quantities become consistent with diffraction data: it may thus be argued that the approach reviewed here is the first one that establishes a direct link between measurements and elements of network theories. Applications for liquid water, simple alcohols, and alcohol–water liquid mixtures demonstrate the usefulness of the aforementioned characteristics. The procedure can readily be applied to more complicated hydrogen-bonded networks, like mixtures of polyols (diols, triols, sugars, etc.) and water, and complex aqueous solutions of even larger molecules (even of proteins).

## Introduction

Hydrogen bonds^1−3^ (HBs) play a most fundamental role in (also in maintaining) our lives: no system of even the faintest biological relevance would be able to exist without this fascinating, yet elusive, intermolecular interaction. For this reason, such systems have been investigated with an outstanding intensity over the past several decades.

In complex biological systems the competition between the hydrophobic and hydrophilic parts of the molecules has a fundamental effect on their properties. Liquid mixtures of simple alcohols (methanol, ethanol, propanol) and water offer a “playground” for studying the complicated structure of hydrogen-bonded networks in relatively simple systems. Alcohol molecules, as previously mentioned, possess both hydrophobic and hydrophilic parts, so their aqueous solutions may be considered as excellent opportunities for scrutinizing how the hydrophobic part of alcohol molecules perturb the H-bonded network formed by water molecules.

In this respect, the knowledge of correlations between atoms within the first coordination shell, and between neighboring coordination shells, as well as over entire hydrogen-bonded aggregates (“clusters” that may percolate, i.e., span the entire system), are all important. Such correlations may all be captured via finding/identifying hydrogen bonds and, later, scrutinizing correlations between hydrogen bonds.

No experiment is able to provide the information mentioned above directly. For this reason, computer simulations are frequently invoked. The resulting 3D particle configurations have proven to be suitable for exploiting achievements of various, seemingly distant, research fields—in our present case, this “distant field” is *network science*.

However, complex studies, involving tools ranging from experiments to elements of network theory, are scarce. Perhaps the works of Dougan et al.^4−8^ are the ones that get the closest to this concept: authors of these studies show that ethanol, like methanol, bipercolates at certain concentrations. Here we wish to briefly describe our own approach that has been developing, step-by-step, during systematic studies of the simplest hydrogen-bonded systems: liquid mixtures of simple alcohols with water.

During the past 5 years, the present authors have considered various alcohol–water mixtures^9−15^ where the focus was put on revealing characteristics of the hydrogen-bonded network. It must be stressed that these^9−15^ works have relied heavily on previous investigations, that had been performed much earlier, over a couple of decades, by various authors (see, e.g., refs (1, 2, 4−8) and references therein). Based on this experience, an approach has crystallized, containing the following steps:

(1) X-ray and/or neutron diffraction experiments are performed on the systems of interest. Here, these systems are alcohol–water liquid mixtures.
(2) Molecular dynamics simulations are performed of the systems of interest.
(3) A consistency check is performed, establishing the quality of agreement between measured and simulated total scattering structure factors. Depending on the quality of agreement, simulated particle arrangements may (or may not) be considered as representations of the real systems.
(4) Provided that the outcome of the consistency check is positive, hydrogen-bonded assemblies are scrutinized within a continuously expanding neighborhood of molecules:
  (4a) Hydrogen bonds are found.
  (4b) Simple descriptors like the number of H-bonds/molecule are determined.
  (4c) H-bond related three-dimensional (3D) distribution functions (see, e.g. refs (16) and (17)) are calculated for the first and second coordination shells of molecules participating in H-bonds.
  (4d) Small cyclic entities, up to 8(−10) members, are identified.
  (4e) Noncyclic assemblies are identified.
  (4f) Clusters of any size are identified, and size distributions are determined, and percolation thresholds can be discussed.
  (4g) Laplace spectra,^18,19^ reflecting genuine cooperative effects that extend over the entire H-bonded network, may be calculated.

In what follows, the above steps will shortly be introduced one-by-one, along with the exhibition of some demonstrative examples from our recent investigations of alcohol–water mixtures.^9−15^ All sets of measurements and computer simulations have been performed as a function of composition *and* temperature: this way, additional aspects of hydrogen bonding can be made visible. Also, this is what makes our recent studies uniquely comprehensive.

## X-ray and Neutron Diffraction Experiments

Neutron and X-ray diffraction are the most important tools for obtaining direct structural information on liquids (including complex ones, such as electrolyte solutions, ionic liquids, aqueous mixtures), see, e.g. refs^20^ and (21). Experimental scattering intensities (as the function of the scattering variable), are directly related to the weighted total radial distribution function, *G*(*r*), via Fourier transformation.

The principal advantage of neutron diffraction, as compared with its X-ray counterpart, arises from the fact that neutrons interact with the atomic nuclei (and not with the electronic cloud, see below the X-ray case), and thus, the scattering interaction is isotropic. Furthermore, neutrons can be sensitive to the isotopic composition of materials: from the point of view of hydrogen bonding, the difference between the scattering powers of hydrogen and deuterium, ^1^H and ^2^H (“H” and “D”), is of primary importance. That is, samples with different isotopic compositions (where the isotopes in question possess markedly different coherent scattering lengths) result in different diffraction patterns, while the underlying structural features remain unchanged. This method is called the isotopic substitution technique. The sensitivity of the final results to details of sample preparation and handling, as well as to the data treatment (normalization, correction term) still remains an open question.^22^ We also note, in passing, that in order to take full advantage of the contrast between H and D, neutron diffraction with polarization analysis is the most advantageous technique (see, e.g., refs (23) and (24)), even though not all difficulties with this approach have been solved satisfactorily as yet.

Concerning X-ray diffraction, the scattering power of an atom is characterized by the form factor, *f*_*α*_(*Q*), that depends significantly on the scattering variable *Q*: *f*_*α*_*(Q)* decreases monotonously with *Q*. *f*_*α*_(0) is equal to the number of electrons of the scattering atom, which also means that the maximum scattering power of a given element (detectable in forward scattering, *Q* = 0) is determined by the number of electrons in its atoms. In contrast to X-rays, neutrons can be scattered equally strongly by light and heavy elements—i.e., hydrogen (both H and D) scatters coherently just as effectively as any heavy atom.

All *X-ray diffraction measurements* reported here have been carried out at the BL04B2 high energy X-ray diffraction beamline of the Japan Synchrotron Radiation Research Institute (SPring-8, Hyogo, Japan).^25^ The beam energy has always been 61.2 keV, which corresponds to a wavelength of 0.203 Å. Diffraction patterns have been recorded in transmission mode, in the horizontal scattering plane, using an array of 6 solid state detectors. In this setup, the available scattering variable (*Q*) range is between 0.16 and 16 Å^–1^. Samples were contained in thin-walled glass capillaries, with an inner diameter of 2 mm and a wall thickness of 0.15 mm. The entire sample environment was under vacuum. To prevent evaporation, the capillaries were sealed with Torr-seal. According to the standard data evaluation protocol at the beamline,^26^ measured intensities are normalized by the incoming beam intensity, and corrected for absorption, polarization, and contributions from the empty capillary. The patterns over the entire *Q*-range are obtained by normalizing and merging each frame, then removing the Compton scattering contributions.

*Neutron diffraction experiments* for our recent studies on alcohol–water mixtures have been performed at the 7C2 diffractometer^27^ of the Laboratoire Léon-Brillouin (LLB Saclay, France), using the standard cryostat available at the beamline. Standard 6 mm vanadium cans were used for containing the liquid samples. The incoming wavelength was 0.72 Å, corresponding to a *Q*-range of 1.06 to 15.7 Å^–1^. Each measurement was controlled by software available at the beamline. The summarized data sets were corrected for efficiency, using a vanadium standard. Intensities from the empty container were also subtracted.

## Molecular Dynamics Simulations

However powerful and exclusive, diffraction experiments on their own are unable to provide the desired detailed picture of the structure—mainly because their primary outcome, the total scattering structure factors (“tssf”), are obtained in the reciprocal space, and in addition, they can only characterize two-body correlations. Thus, many-body correlations, and among them, correlations describing details of hydrogen bonding, remain obscured. This is the reason why we turn to computer simulation methods (for an overview, see ref (28)): they are able to describe our systems in real space, by providing 3D particle configurations. From these particle arrangements, any function that describes many-body correlations can be calculated. Nowadays the most popular branch of simulation methods is molecular dynamics (MD, see ref (28)).

Classical molecular dynamics (MD) simulations have been carried out by using the GROMACS software^29^ (version 2018.2). The Newtonian equations of motion were integrated by the leapfrog algorithm, using a time step of 1 fs. The particle-mesh Ewald algorithm was used for handling long-range electrostatic forces.^30,31^ For each kind of alcohol molecule, the all-atom optimized potentials for liquid simulations (OPLS-AA)^32^ force field was used. Bond lengths were kept fixed by the LINCS algorithm.^33^ Based on results of our recent studies,^10−15^ the TIP4P/2005^34^ and, in some cases, also the SPC/E^35^ water models may be suggested as generally applicable: these can be handled by the SETTLE algorithm.^36^ Typically, several thousand (3000–4000) molecules (with respect to compositions and densities) were placed in cubic boxes, with periodic boundary conditions. Details of the simulations can be found in refs (10−15).

## Consistency Checks between Experiment and Simulations

A key point of the protocol is to establish the extent to which simulated structures may be considered to be valid representations of the real systems. In order to be able to make substantiated statements, the function closest to measured data, the total scattering structure factor, *F*(*Q*), has been considered: experimental *F*^*E*^*(Q)* and simulated *F*^*S*^(*Q*) have been directly compared throughout our recent works.^10−15^

**Figure 1:**
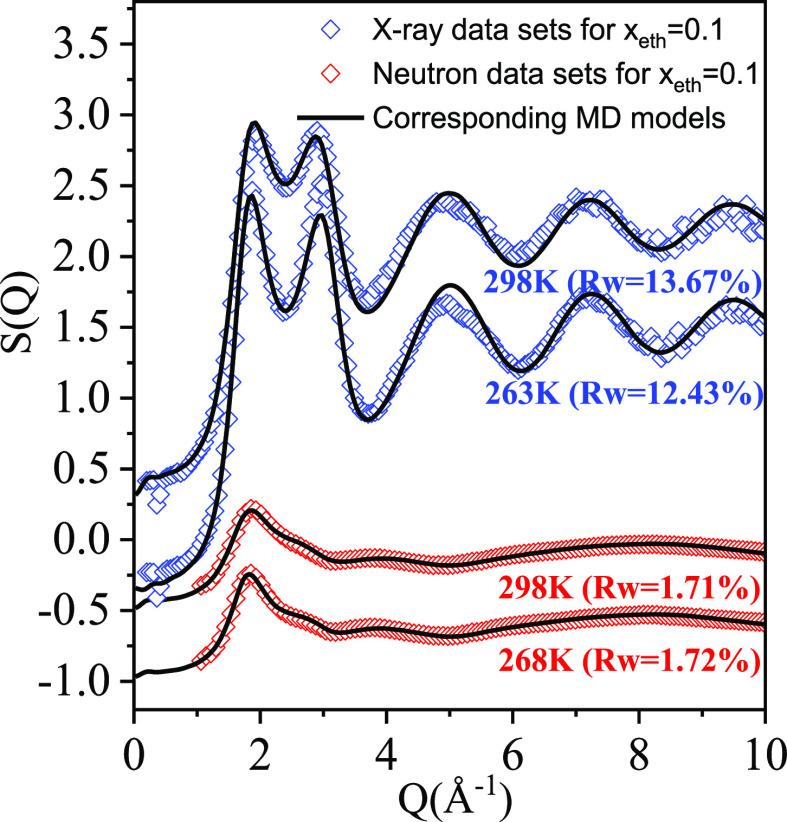
Total scattering structure factors for the mixture with 10 molar % ethanol as a function of temperature. Blue open symbols: X-ray diffraction data;^10^ red open symbols: neutron diffraction data;^10^ solid line: molecular dynamics simulations.^10^ (Curves are shifted for clarity.)

In Figure 1, series of measured and computed *F*(*Q*)s for some of the ethanol–water liquid mixtures considered in ref (10) are contrasted.

By visual inspection, calculated total scattering structure factors are in good agreement with those from diffraction experiments. To quantify this statement, *R*_w_-factors are used:
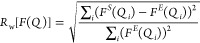
1

*R*_w_-factors provide numerical information on the “closeness” of simulation models to measured data. It is worth noting that these *R*_w_ factors can be considered to be comparable with each other only if applied for identical measurement conditions. As you can see in Figure 1, even for the same systems these values are of different magnitudes for neutron and X-ray diffraction.

Numerical values of *R*_w_-factors have been reported in conjunction with all our studies on liquid alcohol–water mixtures.^9−15^ As is shown in Figure 1, values around 10% represent rather good agreements between simulation and experiment: this level of agreement could be achieved in the majority of cases. In order to give an idea of “good” agreements, *R*_w_-factors corresponding to calculations reported in this work are summarized in Table 1.

**Table 1:** *R*_w_-Factors Corresponding to Calculations Reported in This Work

*R*_w_ (%) ethanol_water (TIP4P/2005) X-ray	298 K	268 K	258 K	253 K	243 K	238 K	233 K	213 K	193 K	ref
10 mol % ethanol	9.76	9.73	14.79	22.71						(10)
20 mol % ethanol	13.51	12.56	15.94	15.59	14.51		21.55			(10)
30 mol % ethanol	13.02	13.27		17.37		14.08				(10)
40 mol % ethanol	19.4						16.2	18.1		(13)
50 mol % ethanol	16						20.3	16.2	16.2	(13)

*R*_w_ (%) isopropanol_water (TIP4P/2005) X-ray	298 K	268 K	263 K	258 K	253 K	243 K	238 K	
5 mol % isopropanol	9.86	14.56						(11)
10 mol % isopropanol	9.61	11.86	11.28					(11)
20 mol % isopropanol	13.38	9.49	10.09	9.68				(11)

*R*_w_ (%) ethanol_water (SPC/E) X-ray	298 K	268 K	258 K	253 K	243 K	238 K	233 K	
10 mol % ethanol	10.88	9.88	13.64	15.15				(10)
20 mol % ethanol	11.11	11.68	12.59	12.14	11.93		17.49	(10)
30 mol % ethanol	14.06	14.91		16.97		15.66		(10)

*R*_w_ (%) isopropanol_water (SPC/E) X-ray	298 K	268 K	263 K	258 K	253 K	243 K	238 K	
5 mol % isopropanol	11.45	20.31						(11)
10 mol % isopropanol	12.67	18.57	16.52					(11)
20 mol % isopropanol	18.79	14.7	15.37	16.85				(11)

*R*_w_ (%) isopropanol_water (TIP4P/2005) neuton	300 K	268 K	263 K	253 K	248 K	243 K	238 K		
30 mol % ethanol	1.5	1.5		1.5	1.6	1.60	1.6		(13)

## Characterization of the Nearby Coordination Spheres via Spatial Distributions

The most common way to characterize short-range order is the calculation of the partial radial distribution functions (rdf), that measure essentially the deviation of the local density of atoms (at a distance “*r*” from the center) from the average (“bulk”) density. However, these functions do not go beyond two-body correlations; that is, they are not capable of characterizing hydrogen bonding in detail. Typical examples for tools that are able to characterize many-body correlations are angular distributions (see, e.g., ref (37)), statistics based on Voronoi polyhedra (see, e.g., ref (38)), or the spherical harmonic coefficients (see, e.g., ref (17)). This latter method has been applied to water and aqueous solution.^17^

Another possible route, that we wish to discuss here in more detail, is the use of various projections of many-body correlation functions. The probability of finding molecules around a central molecule can be presented in 3D: such functions are, for example, *Spatial Distribution Functions* (SDF),^16,17^ that depend on “*r*, *θ*, *φ*” spherical local coordinates, and *Cartesian Spatial Distribution Functions* (C-DSF)^39,40^ that are defined in a Cartesian local coordinate system.

**Figure 2:**
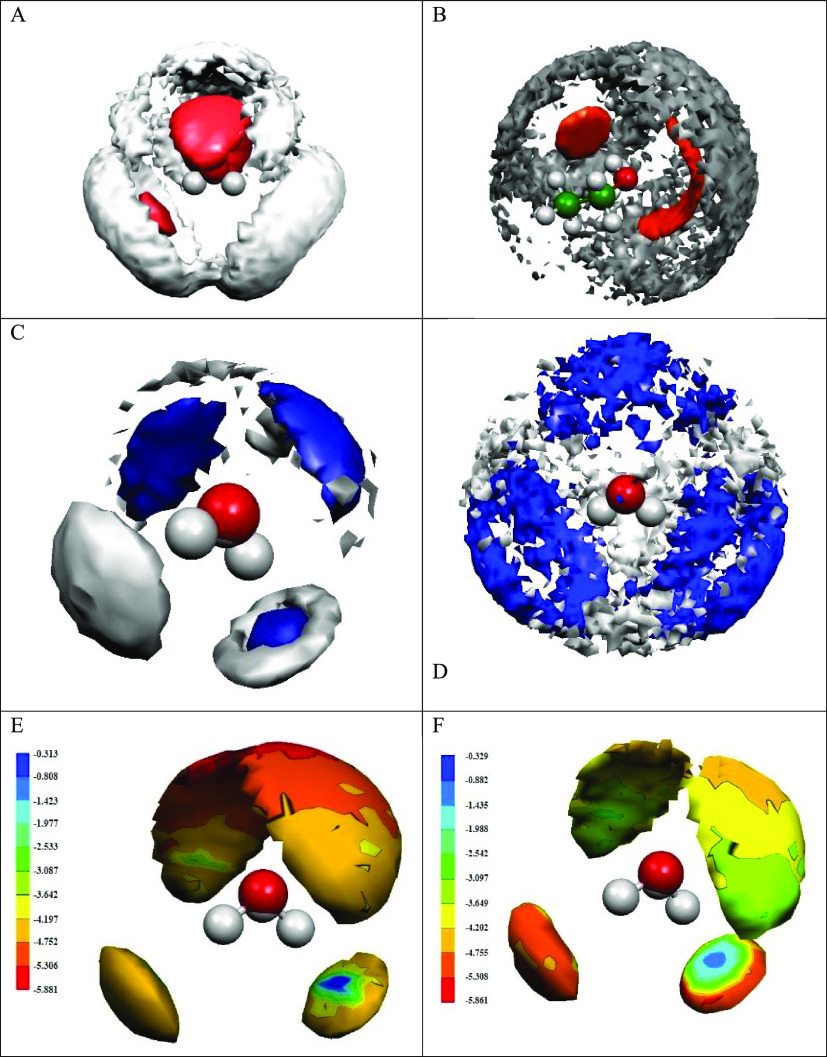
Cartesian spatial distribution functions (SDF) in 40 mol % ethanol–water solutions. (A) First and second shell by C-SDF of water molecules around water molecules at 298 K, (B) first and second shell by C-SDF of ethanol molecules around ethanol molecules at 298 K, (C) differential density C-SDF of water molecules around water molecules (298 K vs 243 K, regarding the first coordination shell), (D) differential density C-SDF of water molecules around water molecules (298 K vs 243 K, regarding the first coordination shell), (E) first shell by C-SDF of water molecules around water molecules, according to water–water energy distribution function at 298 K, (F) first shell by C-SDF of ethanol molecules around water molecules, according to water–ethanol energy distribution function at 298 K. (Red balls: oxygen, gray balls: hydrogen, green balls: carbon. Parts E and F color coding: attractive interaction energies between about −6 (dark red) and −0.5 (dark blue) kcal/mol.)

Such functions provide information about the symmetry and/or directional interactions between a central molecule and the surrounding molecules in the different hydration shells (Figures 2A, 2B). Structural changes caused by temperature differences can also be followed using density difference C-SDF as seen in Figures 2C and 2D. An energy distribution can be superimposed on the C-SDF function with a certain cutoff for the selected shell (Figures 2E and 2F). These examples are just meant to demonstrate the various possible exploitations of C-SDF-s: for a more specific description, see, e.g., ref (11).

## Identification of Hydrogen Bonds

Computational analyses of hydrogen-bonded networks from simulation coordinates require a reasonable definition of a hydrogen bond. Such a definition must take two significant properties of a hydrogen bond into account: its directionality and attractive character. In the literature, several^41−43^ comprehensive investigations can be found that address the influence of various H-bond definitions on calculated topological and dynamical properties. The two most often applied criteria are based on geometric or/and energetic properties of a pair of H-bonded molecules. Purely topological^44^ and continuous^45^ types of H-bond definitions can also be found in the literature.

According to the geometric criterion, the positions of the first minima of the calculated O–O prdf’s and/or the calculated O–H prdf’s, together with the O–H···O or O···O–H angle, are considered. In the energetic criteria, the cutoff energy corresponds to the minimum of the pair energy distribution (−3.0 kcal/mol) of water dimers. It is possible to apply the two definitions together. The correctness of these H-bond definitions was demonstrated by using quantum chemical descriptors.^46^

In our earlier works^9−15^ that the present contribution is based on, two molecules were considered hydrogen bonded to each other if (1) they were found at a distance *r*(O···H) < 2.5 Å and (2) the interaction energy was less than −3.0 kcal/mol. In all cases pure geometric criteria (*r*(O···H) < 2.5 Å, H–O···O angle <30°) were also considered. It has been found that the exact H-bond definition applied has not resulted in significant differences concerning the main conclusions.

## H-Bond Numbers and Their Distributions

H-bonded neighbors of molecules can be quantified by applying the H-bond definition(s) introduced above. The resulting average number of H-bonds can be calculated for the entire system, as well as for its subsystems, such as water–water, water–alcohol, and alcohol–alcohol. Moreover, molecules can be classified on the basis of the number of hydrogen bonds they take part in as H-donors (*n*_D_ = 0, 1 for alcohol and *n*_D_ = 0, 1, or 2 for water molecules) and H-acceptors (for example *n*_A_ = 0, 1, 2 for alcohol and *n*_A_ = 0, 1, 2 (, 3) for water molecules). Molecules may thus be tagged as (*n*_D_D:*n*_A_A).

**Figure 3:**
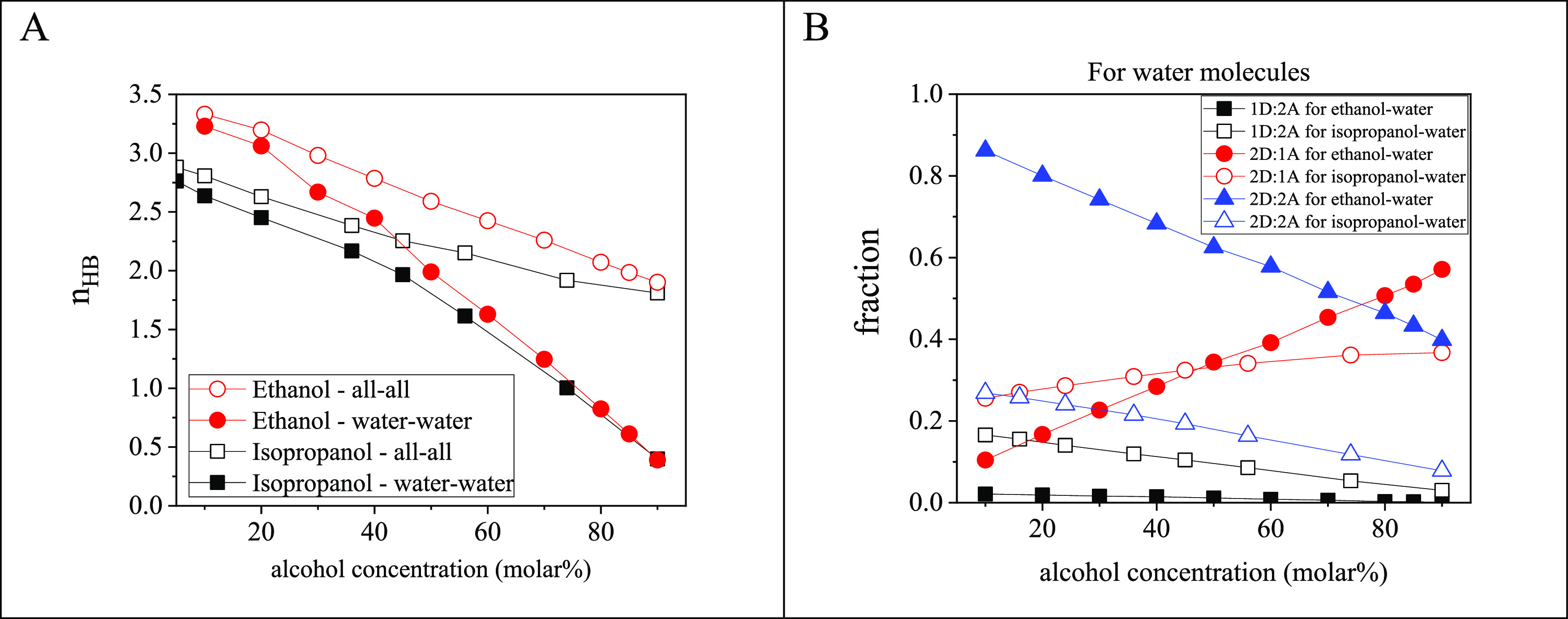
(A) Average H-bond numbers, *n*_HB_, considering each molecule, regardless of their types, and considering water molecules around water molecules only. (B) Donor and acceptor sites of water molecules as a function of alcohol concentration.

The average H-bond numbers are shown in Figure 3A for ethanol–water and isopropanol-water mixtures at 298 K as a function of the alcohol content. In both cases, independently of the type of molecules involved in H-bonding, *n*_HB_ decreases with the water content. Over the whole concentration range, molecules in ethanol–water systems form more H-bonds than they do in isopropanol–water mixtures. Figure 3B shows the decomposition of H-bond numbers according to donor and acceptor roles for three typical cases, namely for water molecules of types 1D:2A, 2D:1A, and 2D:2A. With increasing alcohol content, the ratios of 2D:2A and 1D:2A molecules decrease, while the ratio of 2D:1A molecules increases in both alcohol–water mixtures. In the water rich region, the fraction of water molecules with fully occupied (acceptor and donor) H-bonding sites is much larger in ethanol–water than in isopropanol–water systems, due to the different alkyl chain sizes.

## Cluster Size Distributions and Percolation Analyses

Aqueous alcohol solutions can be considered as networks of molecules whose neighboring members (“nodes”) are connected to each other via H-bonds. For determining the percolation transition in such systems, various descriptors may be used.

The *cluster size distribution*, *P*(*n*_c_) can be approximated theoretically by the following formula:
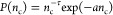
2where *a* is a constant, *n*_c_ is the cluster size, and τ depends on the dimensionality of the system.^47^ In simulation boxes, where particle coordinates are present, more practical considerations are available. Two molecules are regarded as belonging to the same cluster if a connection, via a chain of hydrogen bonds, can be found between them. The system is said to be percolating if the number of molecules in the largest cluster is on the order of the system size. The cluster size distribution can be given by *P*(*n*_c_) = *n*_c_^–2.19^ for random percolation on a 3D cubic lattice. The percolation transition can be detected by comparing the calculated cluster size distribution function of the simulated system with that obtained for a random system.^47−50^

In addition, the following descriptors, that originate in network science,^51−55^ can also be used for finding the appropriate value of the percolation threshold:
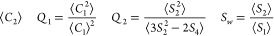
where *C*_1_ and *C*_2_ are the sizes of the largest and the second largest cluster, respectively. *S*_1_, *S*_2_, and *S*_4_ are the first, second, and fourth moments of the cluster size distribution. When calculating *S*_w_, the largest cluster is excluded from the calculation.

**Figure 4:**
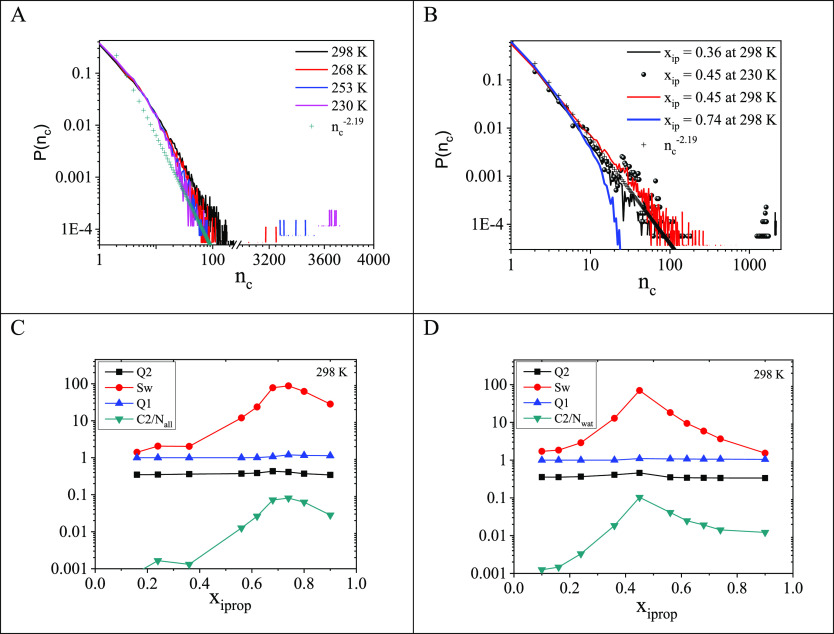
(A) Cluster size distributions for the 74 molar % isopropanol aqueous solution at different temperatures. (B) Cluster size distributions for *x*_iprop_ = 0.36 and 0.45 in isopropanol aqueous solutions. (C) The ⟨*C*_2_⟩, *S*_w_, *Q*_1_, and *Q*_2_ quantities for the characterization of the percolation transition in isopropanol aqueous solution as a function of concentration at 298 K. (D) The ⟨*C*_2_⟩, *S*_w_, *Q*_1_, and *Q*_2_ quantities for the characterization of the percolation transition for the water–water subsystems in isopropanol aqueous solution as a function of concentration at 298 K.

Figure 4A shows that in the 74 molar % isopropanol aqueous solution at 230, 253, and 286 K the system is percolated. However, at 298 K, on approaching the threshold value, the determination of the percolation threshold may become uncertain. The same statement can be made based on Figure 4B, where, between *x*_iprop_ = 0.36 and 0.45, *P*(*n*_c_) functions run very much together. It would be advisible to take other descriptors into account, as defined above: these are shown in Figure 4C and D. These descriptors clearly prove the existence of a percolation threshold at their maximum values.

## Cyclic and Noncyclic Entities

Concerning the topology of hydrogen-bonded aggregates in alcohol—water liquid mixtures, two types of clusters can be identified.^10−14,56−60^ In pure alcohols, very few rings can be detected: mostly longer and shorter chains, with various branching positions, exist. In liquid water the network contains mainly cyclic units. In mixtures of alcohols and water, the distribution of these basic types depends on parameters such as the molecular size of the alcohol, composition, and temperature.

The frequently used “closed path statistics” type analysis was introduced by King^61,62^ and has been developed significantly over time.^63^ In our works the primitive ring criterion^64,65^ was applied: a ring is called “primitive” if it cannot be decomposed into smaller rings.

**Figure 5:**
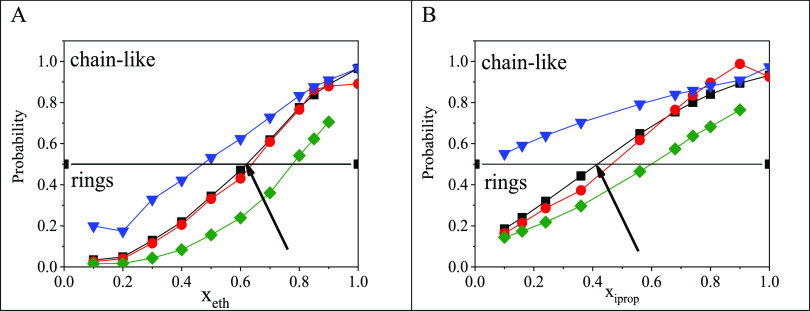
Characteristic properties concerning the existence of cyclic and noncyclic entities in water–ethanol (A) and water–isopropanol (B) mixtures as a function of alcohol concentration. Black squares: molecules outside of cyclic entities; red circles: H-bonds outside of cyclic entities; blue down triangles: ethanol (A) or isopropanol (B) molecules outside of cyclic entities, olive diamonds: water molecules outside of cyclic entities.

Following this definition, the mixtures considered here can be characterized by, for instance, the ratio of molecules *not* participating in cyclic entities (Figure 5). This quantity can also be calculated for the subsystems, namely the number of alcohol molecules *not* participating in cycles divided by the total number of alcohol molecules, and analogously, the same for water molecules. Furthermore, the calculation can be applied for the H-bonds themselves, thus yielding the ratio of H-bonds found *outside* of cyclic entities. If chain-like structures are the majority in a given system, then these quantities are above 0.5, while below this mostly rings can be found. This threshold can be observed at *x*_eth_ = 0.62 and *x*_iprop_ = 0.42. Ethanol molecules prefer to form chains at a higher ethanol content (above *x*_eth_ = 0.47), while isopropanol molecules prefer to be members of chain-like structures over the entire concentration range. On the other hand, water molecules in both systems are found mostly in rings, even in the alcohol rich region (below *x*_eth_ = 0.78 for ethanol–water and below *x*_iprop_ = 0.6 for isopropanol–water mixtures).

**Figure 6:**
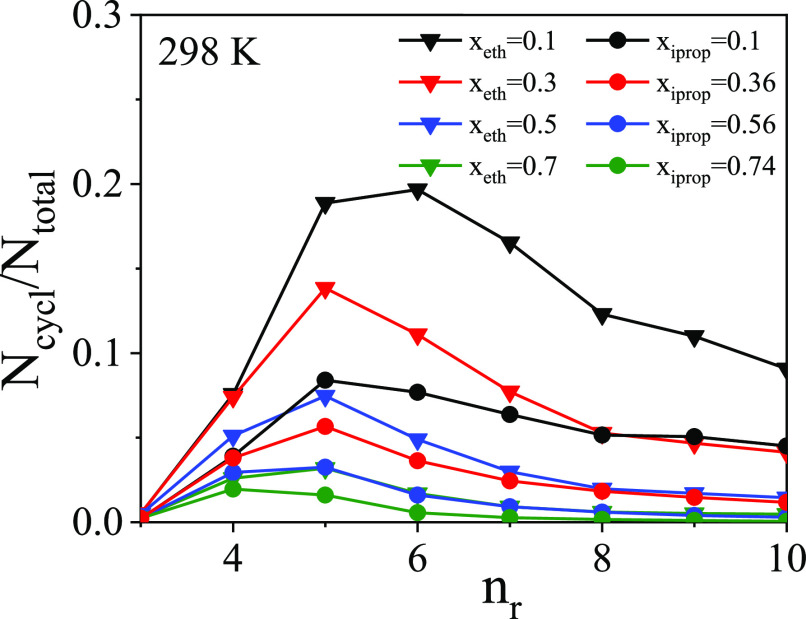
Normalized ring size distributions for ethanol–water (triangles) and isopropanol–water (circles) mixtures at 298 K.

Figure 6 shows normalized ring size distributions for ethanol–water and isopropanol–water mixtures at 298 K, as a function of concentration. At the lowest ethanol concentration studied (*x*_eth_ = 0.1), six-membered rings are the most populous, similar to what was found^9^ for pure water. However, for alcohols with longer chains (namely, isopropanol in the present study), five-membered rings are preferred (to six-membered ones) already at low concentrations (*x*_iprop_ = 0.1). With an increasing alcohol content, five-membered rings become dominant in both systems in question. Above a certain concentration of alcohol molecules (circa *x*_alcohol_ = 0.7), four-membered rings appear. Note, however, that in this concentration range the number of rings is very small.

## Laplace Spectra

Finally, a tool for revealing genuinely cooperative properties is touched upon. The Laplacian matrix of a graph and its eigenvalues can be used in various areas of mathematics, mainly discrete mathematics and combinatorial optimization, as well as for interpreting several physical and chemical problems.^18,19,66,67^ For example, the second smallest eigenvalue of the Laplacian matrix (also called “spectral gap”) and the corresponding theory connecting to the Cheeger inequality^68,69^ is broadly considered to be a critical parameter that influences the stability and robustness properties of dynamical systems that are implemented over information networks. The Laplace matrix can be defined as follows:^18,19,66,67^
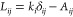
3where *k*_*i*_ is the number of (hydrogen) bonded neighbors of molecule “*i*”; *δ*_*ij*_ is the Kronecker delta function; and *A*_*ij*_ = 1 if a bond exists between nodes *i* and *j*.

It is known that the Laplacian matrix is positive semidefinite and has nonnegative eigenvalues.^18,19^ It can be proven that the multiplicity of eigenvalue 0 (it always exists) of matrix L is equal to the different connected components in the graph and the corresponding eigenvectors characterize the connected components.

**Figure 7:**
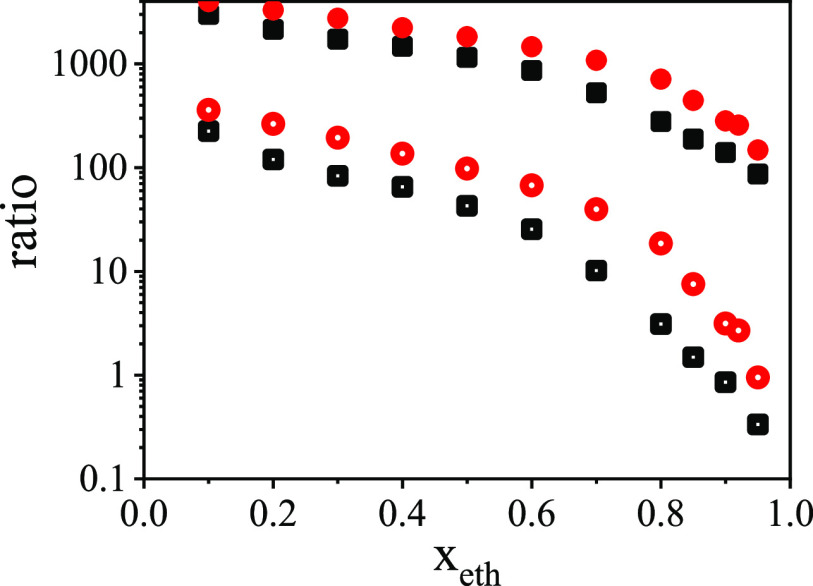
Indicators of the “stability” of H-bond networks in ethanol–water mixtures at 298 K (open symbols) and at 233 K (solid symbols). Red symbols: upper limits, black symbols: lower limits (based on the Cheeger-inequality.^68,69^

As a demonstration, in Figure 7 we would like to provide some indicator for the “stability” of H-bonded networks found in ethanol–water mixtures, as a function of temperature.^13^ These data were calculated by using the Cheeger inequality^68,69^ that provides upper and lower limits on the stability of an H-bonding network. It can be seen that the stability of the hydrogen bond network decreases significantly with increasing ethanol concentration.

These results can be used as new descriptors that can provide information on the percolation transition. Some other applications of the network theory for investigating H-bond networks using adjacency matrix in liquids can be found in refs (70−72).

## Summary and Future Perspectives

A number of simple alcohol–water mixtures have recently been studied according to a novel “protocol” over the past few years.^9−15^ During, and as a result of, these systematic investigations, an approach has been evolved that aims at a better understanding of hydrogen-bonded networks. In this Perspective, elements of the protocol have been listed and summarized briefly, from diffraction experiments to as far as network theories. The usefulness of the approach has been demonstrated by flashing results on ethanol–water and isopropanol–water liquid mixtures, like the determination of (the composition and temperature dependence of) the size distribution of hydrogen-bonded aggregates, percolation transition points, and size distribution of small cyclic entities. We stress that, as a result of applying the novel protocol, these latter, rather abstract, quantities are always consistent with experimental (diffraction) data: it may thus be argued that the approach reviewed here is the first one that establishes a direct link between measurements and elements of network theories.

The protocol can readily be applied for more complicated hydrogen-bonded networks, like mixtures of polyols (diols, triols, sugars, etc.) and water, and complex aqueous solutions of even larger molecules (even of proteins). It is important to realize that in these more complicated cases, as a new challenge, we have to take into account the “surface” of the molecules. Here, van der Waals and C–H···O type interactions, or in other words hydrophobic contributions, become comparable with hydrophilic (H-bonding) ones. A possible approach would be the identification of hydrophobic and hydrophilic disjunct surfaces: these sites help to recognize the active parts of enzymes, as well as the minor and major grooves of the DNA helix structures. Some attempts are described in the literature^73−75^ in which the structure of proteins and the hydrate structure formed around them are investigated with the help of small- and wide-angle X-ray diffraction experiments. We believe that a logical extension of our approach would be a joint use of data from both small- and wide-angle diffraction experiments.
